# Eating disorder symptom trajectories in adolescence: effects of time, participant sex, and early adolescent depressive symptoms

**DOI:** 10.1186/2050-2974-1-32

**Published:** 2013-08-20

**Authors:** Karina L Allen, Ross D Crosby, Wendy H Oddy, Susan M Byrne

**Affiliations:** 1 Telethon Institute for Child Health Research, Centre for Child Health Research, The University of Western Australia, PO Box 855, West Perth, WA, Western Australia; 2 School of Psychology, The University of Western Australia, Crawley, Western Australia; 3 Department of Clinical Neuroscience, University of North Dakota School of Medicine and Health Sciences, Fargo, North Dakota, USA; 4 Department of Biostatistics, Neuropsychiatric Research Institute, Fargo, North Dakota, USA

**Keywords:** Eating disorders, Binge eating, Purging, Fasting, Dieting, Exercise, Adolescence, Trajectory, Raine study

## Abstract

**Background:**

Adolescence is a period of developmental risk for eating disorders and eating disorder symptoms. This study aimed to describe the prevalence and trajectory of five core eating disorder behaviours (binge eating, purging, fasting, following strict dietary rules, and hard exercise for weight control) and a continuous index of dietary restraint and eating, weight and shape concerns, in a cohort of male and female adolescents followed from 14 to 20 years. It also aimed to determine the effect of early adolescent depressive symptoms on the prevalence and trajectory of these different eating disorder symptoms. Participants (N = 1,383; 49% male) were drawn from the Western Australian Pregnancy Cohort (Raine) Study, a prospective cohort study that has followed participants from pre-birth to age 20 years. An adapted version of the Eating Disorder Examination-Questionnaire was used to assess eating disorder symptoms at ages 14, 17 and 20 years. The Beck Depression Inventory for Youth was used to assess depressive symptoms at age 14. Longitudinal changes in the prevalence of eating disorder symptoms were tested using generalised estimating equations and linear mixed models.

**Results:**

Symptom trajectories varied according to the eating disorder symptom studied, participant sex, and the presence of depressive symptoms in early adolescence. For males, eating disorder symptoms tended to be stable (for purging, fasting and hard exercise) or decreasing (for binge eating and global symptom scores) from 14 to 17 years, and then stable to 20 years. For females, fasting and global symptom scores increased from age 14 to peak in prevalence at age 17. Rates of binge eating in females were stable from age 14 to age 17 and increased significantly thereafter, whilst rates of purging and hard exercise increased from age 14 to age 17, and then remained elevated through to age 20. Depressive symptoms at age 14 impacted on eating disorder symptom trajectories in females, but not in males.

**Conclusions:**

Prevention, screening and intervention initiatives for adolescent eating disorders need to be tailored to gender and age. Purging behaviour appears to be an important target for work with early to middle adolescent females.

## Background

Adolescence is regarded as a period of developmental risk for eating disorders [1,2] and eating disorder symptoms [3-5]. In general, eating disorder symptoms increase from 14–16 years in females [3,4], with peaks in binge eating onset at 16–17 years and purging onset at 17–18 years [1,6]. Few studies have considered the developmental trajectory of eating disorder symptoms in males, but available data suggest that boys may show reductions in eating pathology from early adolescence to mid adolescence, and then experience an increase in symptoms in late adolescence or early adulthood [3,5,7,8]. Eating disorder symptoms are associated with impairments in quality of life even when full criteria for an eating disorder are not met [9-11]. This makes it important that they are considered in research on the epidemiology and consequences of eating disorder psychopathology.

Studies have varied considerably in how eating disorder symptoms are conceptualised and assessed. Some research groups have used continuous questionnaire scores, which reflect a global index of cognitive and behavioural symptoms (such as dietary restraint and concerns about eating, weight and shape) and/or tendencies towards binge eating and purging (e.g., [3,7]). Others have assessed for discrete eating disorder behaviours, such as strict dieting, binge eating, or purging (e.g., [6,8]). In contrast to several large-scale cross-sectional projects [11,12], longitudinal studies in this area have not tended to assess for the full range of behaviours that may inform eating disorder diagnostic decisions, such as binge eating, purging, fasting and driven exercise in a single sample followed over time. An exception is in the study by Neumark-Sztainer et al. [5], where dieting, unhealthy weight control behaviours (defined as fasting, eating very little, using food substitutes, skipping meals or smoking cigarettes), extreme weight control behaviours (defined as diet pills, vomiting, laxatives or diuretics), and binge eating were assessed prospectively for male (n = 1030) and female (n = 1257) participants. Symptom trajectories varied according to the symptom in question, with marked increases being observed in the prevalence of extreme weight control behaviours across adolescence, but relative stability being observed in dieting behaviours [5]. Trajectories also varied for males and females, with symptom increases in middle adolescent females contrasting with symptom decreases in middle adolescent males. These findings highlight the importance of distinguishing between different symptom sets, and between male and female participants, when assessing eating disorder symptoms over time. To achieve this, longitudinal studies need to ensure that eating disorder assessment methods are broad enough to distinguish between different behaviours.

Eating disorders and eating disorder symptoms are strongly associated with other mental health difficulties, including depression, anxiety, substance misuse, and personality disorders [13-16]. These associations are particularly well documented for depression [14,16] and it has been established that depression and eating disorder symptoms show a bi-directional relationship, with each predicting increases in the other over time [17-19]. Most of this research has been conducted with females [17-20], but Sonneville and colleagues found significant associations between binge eating behaviour and later onset of depressive symptoms in male (n = 7843) and female (n = 9039) participants followed from adolescence to young adulthood in the Growing Up Today Study (GUTS) [8]. Conversely, Gardner and colleagues found that depression predicted increases in eating disorder symptoms in 6- to 14-year-old boys (n = 112) and girls (n = 104) over a 3-year period [21]. The dual-pathway model of bulimia nervosa (BN) also positions depressive symptoms as one pathway to binge eating and purging [22], and this model has been validated in male and female participants [23-25].

To date, studies on depression and eating pathology have tended to focus on binge eating and purging behaviours (e.g., [6,8]), or to use a global index of eating disorder symptoms (e.g., [17,19,26]). It is not clear whether depression also relates to other eating disorder symptoms, such as strict dietary restraint, purging without binge eating, or driven exercise for weight control. If depression predicts increases in some eating disorder symptoms but not others, it may be possible to tailor prevention programmes accordingly. Similarly, if these relationships differ across males and females, prevention efforts should be modified according to gender. The high prevalence of depressive symptoms in adolescence [27] makes delineation of these effects important, as results may facilitate targeted eating disorder screening, prevention and intervention work in adolescent populations.

Given the above, the current study had two aims:

1. To describe the prevalence and trajectory of five core eating disorder behaviours (binge eating, purging, fasting, following strict dietary rules, and hard exercise for weight control), and a continuous global index of eating pathology, in a cohort of male and female adolescents followed from 14 to 20 years.

2. To determine the effect of early adolescent depressive symptoms on the prevalence and trajectory of these different eating disorder symptoms over the 6-year time period, for males and females separately.

## Methods

### Design and participants

Participants were drawn from the Western Australian Pregnancy Cohort (Raine) Study, a prospective cohort study of 2,868 live birth babies followed to 20 years of age.

Full details regarding the Raine Study have been published previously [28,29]. In summary, 2,900 women were recruited from the antenatal booking clinics at King Edward Memorial Hospital for women (KEMH), the only public maternity hospital in Western Australia, between May 1989 and November 1991. Of the 2,900 women enrolled, 2,804 delivered live birth babies. Due to 64 multiple births, the initial cohort included 2,868 children. These children were assessed at birth and ages 1, 2, 3, 5, 8, 10, 14, 17 and 20 years.

This study focused on the 14, 17 and 20-year follow-ups, when detailed eating disorder data were collected. We used data from the earlier assessments to consider differences between participants included in this study and those lost to follow-up prior to adolescence. Eating disorder data were available for 1,598 participants at age 14 (56% of the original 2,868 births), 1,242 participants at age 17 (43%), and 1,243 participants at age 20 (43%). Analyses make use of participants with data at age 14 and at least one of the subsequent follow-ups, giving an effective sample size of 1,383 (49% male). This sample of 1,383 participants represents 48% of the original 2,868 births, 59% of the participants who were eligible for participation in the 14 through 20-year assessments (i.e., not deceased or lost to follow-up prior to age 14; N = 2,328), and 76% of the participants who completed at least one of the 14 through 20-year assessments (N = 1,878). Regarding attrition prior to age 14, 30 participants died in childhood (1% of the original 2,868 births), 162 were unable to be contacted (6%), and 348 had withdrawn (12%), resulting in a total possible sample of 2,328 adolescent participants (81%).

The mean age of the participants in this study was 14.01 years (SD = 0.19, range = 13.00–15.08) at the 14-year assessment, 16.92 years (SD = 0.24, range = 15.0–18.2) at the 17-year assessment, and 20.01 years (SD = 0.44, range = 19.00–22.08) at the 20-year assessment.

### Procedure

Questionnaire packages were posted to adolescents at the 14, 17 and 20-year assessments, for at-home completion prior to a face-to-face assessment session. Height and weight were measured objectively during the face-to-face assessment.

All data collection occurred in accordance with Australian National Health and Medical Research Council Guidelines for Ethical Conduct in Human Research and was approved by the ethics committees of KEMH, Princess Margaret Hospital for Children, and the University of Western Australia.

### Measures

#### Eating disorder symptoms at 14, 17 and 20 years

Eating disorder symptoms were assessed using 24 self-report items adapted from the Child Eating Disorder Examination (ChEDE) [30] and Eating Disorder Examination-Questionnaire (EDE-Q) [31]. These 24 items were self-report, as per the EDE-Q, but language was simplified or clarified when there was the possibility of confusion for 14-year-old adolescents. Response options were also simplified, so that the same four response options were used for all items: “not at all” (0); “some of the time (once per week / a few times a month)” (1); “a lot of the time (a few times a week)” (2); and “most of the time (every day or nearly every day)” (3). Questions referred to the previous month and the same items were used at all assessment points. Participants were asked to be conservative in their answers if they were unsure of the frequency of their behaviours. There is good evidence for the validity of self-report eating disorder assessment [31-36], including self-report assessment in adolescence [37].

Five categorical variables were computed to indicate the presence or absence of core eating disorder behaviours over the previous month: objective binge eating (eating an objectively large amount of food and feeling out of control of one’s eating), purging (self-induced vomiting and/or laxative misuse), hard exercise specifically for weight control, fasting (not eating for 8 or more waking hours), and attempts to follow strict dietary rules. Behaviours were coded as present if they occurred at least “some of the time (once per week / a few times per month)”. This frequency criterion is consistent with the requirements of DSM-5, which requires weekly binge eating / purging for diagnoses of bulimia nervosa and binge eating disorder [38]. A continuous, global index of eating disorder symptoms was also calculated by taking the mean of the items (n = 18) relating to dietary restraint and eating, weight and shape concern. Distinctions were not made between restraint and eating/weight/shape concerns, or between general weight and shape concerns and the over-evaluation of weight and shape, because of the high degree of correlation between these symptoms and their similar trajectories over time. Alpha coefficients for this global index were .90, .93 and .91, at the 14, 17 and 20-year assessments respectively.

Additional details on the eating disorder items have also been provided previously [29] and a copy of the questionnaire is provided in Appendix A.

#### Depressive symptoms at 14 years

Depressive symptoms at age 14 were assessed using the Beck Depression Inventory for Youth (BDI-Y) [39]. The BDI-Y is an adolescent adaptation of the adult Beck Depression Inventory-2 [40] and has excellent psychometric properties [39,41]. The possible score range is from 0 to 63. The alpha coefficient in this sample at age 14 was .97. Scores on the BDI-Y were stratified according to recommended cut-points for the BDI-Y in early adolescence [39], to give a group with scores in the normal range (score ≤16) and a group with scores suggestive of at least mild depressive symptoms (score > 17).

#### Covariates

Family income and adolescent body mass index (BMI) were included as covariates in all analyses. Family income was reported by parents at the 14, 17 and 20-year assessments and dichotomised into low vs. medium-high income categories, where ‘low’ income included the lowest two Australian income quintiles and captured 15 - 20% of the sample at each assessment point. Adolescent height and weight measurements were taken by a trained researcher at each assessment point and used to calculate BMI according to the standard formula of weight (kg) / height (m)^2^.

Parents also reported on family (e.g., family income, employment status, marital status), parent (e.g., parent physical and mental health) and child (e.g., child mental health) characteristics at the 5, 8 and 10-year assessments. These data were used in preliminary analyses comparing the current sample to Raine Study participants lost to follow-up.

### Statistical analysis

All analyses were conducted in SPSS Statistics Version 19.

#### Preliminary analyses

Participants included in this study (n = 1,383) were compared to Raine Study participants lost to follow-up before adolescence (n = 961). Participants were compared on family, parent and psychosocial variables at ages 5, 8 and 10 years, using independent-samples *t-*tests (continuous variables) and Chi square tests (categorical variables). The same comparisons were conducted across participants included in this study and those who completed only one adolescent assessment (n = 495), and adolescent eating disorder symptoms were also compared across participants included in this study and those who took part in only one adolescent assessment.

After data screening, imputation techniques were used to impute missing eating disorder data for participants who completed two out of three adolescent assessments. Multiple imputation and EM estimation using maximum likelihood were both trialled, using established principles and techniques [42-44]. EM imputation was retained as providing the most appropriate imputation outcomes. Additional details are provided under Results, below.

#### Core analyses

Longitudinal changes in the prevalence of eating disorder behaviours were tested using generalised estimating equations (GEE) [45], which account for correlations within individuals over time. Initially, base models were run to determine if the prevalence of eating disorder symptoms varied significantly over time. Logistic binomial models were specified with a main effect of time, for each symptom. An independent working correlation model was used [46]. Subsequently, models were re-run to examine the predictive effects of 14-year depressive symptoms on changes in eating disorder symptoms over adolescence. These explanatory models were specified with a main effect of time, a main effect of 14-year depression status, and an interaction effect between time and depression. The categorical interaction term was created using three levels of time (1 = age 14, 2 = age 17, 3 = age 20) and two levels of depression (0 = no marked depression at age 14, 1 = pronounced depressive symptoms at age 14).

Longitudinal changes in global eating disorder symptoms scores were examined using linear mixed models. Again, a base model was specified to determine if scores varied significantly over time. Subsequently, time and 14-year depression status were specified as main effects, along with a time × depression interaction.

Models were adjusted for family income and adolescent BMI, and run separately for male and female participants.

## Results

### Preliminary analyses

Raine Study participants lost to follow-up before adolescence (n = 961) were significantly more likely to be from single-parent families at ages 5 (*Χ*^2^ [1] = 17.13, *p* < .001), 8 (*Χ*^2^ [1] = 23.29, *p* < .001), and 10 (*Χ*^2^ [1] = 10.51, *p* < .001) years; were significantly less likely to have employed parents at ages 5 (*Χ*^2^ [1] = 15.51, *p <* .001), 8 (*Χ*^2^ [1] = 13.96, *p* < .001), and 10 (*Χ*^2^ [1] = 24.19, *p* < .001) years; had significantly lower family incomes at ages 5 (*t* [1724] = 5.10, *p* < .001), 8 (*t* [1636] = 5.38, *p* < .001), and 10 (*t* [1578] = 4.67, *p* < .001) years; and had significantly higher CBCL Externalising Problem scores at ages 5 (*t* [1738] = 2.76, *p* = .007), 8 (*t* [1656] = 4.22, *p* < .001), and 10 (*t* [1610] = 2.94, *p* = .003) years, when compared to participants included in this study (n = 1383). These findings are consistent with the tendency for socially disadvantaged families to be lost to follow-up over time [47], and are considered in the Discussion.

Raine Study participants who completed only one adolescent assessment (n = 495) were also significantly more likely to be from single-parent families at age 8 (*Χ*^2^ [1] = 4.60, *p* = .032), but not at ages 5 (*p* = .295) or 10 (*p* = .256) years; were significantly less likely to have employed parents at age 8 (*Χ*^2^ [1] = 8.48, *p* = .004), but not at ages 5 (*p* = .072) or 10 (*p* = .379) years; had significantly lower family incomes at ages 5 (*t* [1696] = 3.28, *p* = .001), 8 (*t* [1663] = 4.10, *p* < .001), and 10 (*t* [1676] = 3.54, *p* < .001) years; and had significantly higher CBCL Externalising Problem scores at ages 5 (*t* [1705] = 2.66, *p* = .008), 8 (*t* [1680] = 4.31, *p* < .001), and 10 (*t* [1704] = 3.14, *p* = .002) years, compared to participants included in this study (n = 1383). There were no significant between-group differences in eating disorder symptom scores or the prevalence of categorical eating disorder behaviours (*p*s = .187 - .986) across participants who completed one adolescent assessment and those who completed two or more.

For participants who completed two of the three adolescent assessments, there was no evidence to suggest that missing eating disorder data were not missing at random (Little’s MCAR test *Χ*^2^ [1399] = 1376, *p* = .664). Nonetheless, a conservative approach was taken by imputing data prior to analysis, rather than relying on the GEE procedure, which would require data to be missing completely at random in order for reliable estimates to be obtained [48]. Data were imputed for 281 participants in total, with 141 participants at age 17 and 140 participants at age 20. Multiple imputation was conducted using eating disorder variables, BMI, depressive symptom scores and CBCL Internalising and Externalising Problem scores as predictors in the imputation model (in varying combinations ranging from few predictors to many predictors) [42]. However, this imputation approach produced marked over-estimates for rare behaviours (i.e., vomiting), irrespective of the particular combination of predictor variables. When EM imputation with maximum likelihood was conducted [43], this over-estimation did not occur. Estimated means and standard deviations for the original and EM eating disorder data were highly similar, as were symptom prevalence rates and associations between eating disorder symptoms and depressive symptoms. Thus, EM imputation was used in preference to multiple imputation. All subsequent analyses make use of the full, imputed data set.

Scores on the BDI-Y ranged from 0 to 34 for boys (M[SD] = 5.06 [5.32]) and from 0 to 50 for girls (M[SD] = 7.88 [7.80]). The proportion of participants classified as having at least mild depressive symptoms (score > 17) was 4.0% (n = 26) for boys and 12.5% (n = 83) for girls, equating to 7.9% of the sample overall (n = 109). The BDI-Y was not completed by 73 participants (36 boys and 37 girls), giving a sample size of 1310 (49% male) for analyses on the effects of depressive symptoms on eating disorder trajectories.

### Changes in eating disorder symptoms over time

Mean global eating disorder symptom scores and prevalence rates for categorical eating disorder symptoms are shown in Table 1 (boys) and Table 2 (girls).

**Table 1:** Eating disorder symptoms and body mass index by time, for male participants (n = 680)

	**14 yrs**	**17 yrs**	**20 yrs**	***p***
Body mass index (M [SD])	20.91_a_ (3.91)	22.74_b_ (4.17)	24.45_c_ (4.59)	<.001
Global symptoms (M [SD])	0.33_a_ (0.35)	0.30_b_ (0.40)	0.31_b_ (0.38)	<.001
Objective binge eating (% yes)	23.6%_a_ (n = 160)	15.0%_b_ (n = 102)	17.2%_b_ (n = 117)	<.001
Purging (% yes)	1.2% (n = 8)	2.5% (n = 17)	2.7% (n = 18)	.123
Fasting (% yes)	9.7% (n = 66)	8.5% (n = 58)	8.1% (n = 55)	.502
Dietary rules (% yes)	21.4%_a_ (n = 145)	16.2%_b_ (n = 110)	21.1%_a_ (n = 143)	<.001
Hard exercise (% yes)	49.0% (n = 333)	46.5% (n = 316)	50.8% (n = 345)	.500

*Note.* Global symptoms refer to a global index of dietary restraint and eating, weight and shape concerns.

*p* values and column subscripts denote significant differences over time, as identified through generalised estimating equations or linear mixed models. Columns with different subscripts differ significantly at *p* < .05.

*a,b,c* indicate significant differences over time.

**Table 2:** Eating disorder symptoms and body mass index by time, for female participants (n = 703)

	**14 yrs**	**17 yrs**	**20 yrs**	***p***
Body mass index (M [SD])	21.49 (4.16)_a_	23.11 (4.51)_b_	24.37 (5.21)_c_	<.001
Global symptoms (M [SD])	0.61 (0.54)_a_	0.82 (0.64)_b_	0.66 (0.50)_a_	<.001
Objective binge eating (% yes)	38.5% (n = 271)_a_	36.8% (n = 259)	43.4% (n = 305)_b_	<.001
Purging (% yes)	3.7% (n = 26)_a_	13.5% (n = 95)_b_	14.0% (n = 98)_b_	<.001
Fasting (% yes)	16.4% (n = 115)_a_	26.5% (n = 186)_b_	19.5% (n = 137)_a_	<.001
Dietary rules (% yes)	33.6% (n = 236)	35.0% (n = 246)	41.0% (n = 288)	.063
Hard exercise (% yes)	56.8% (n = 399)_a_	64.2% (n = 451)_b_	69.4% (n = 488)_b_	<.001

*Note.* Global symptoms refer to a global index of dietary restraint and eating, weight and shape concerns.

*p* values and column subscripts denote significant differences over time, as identified through generalised estimating equations or linear mixed models. Columns with different subscripts differ significantly at *p* < .05.

*a,b,c* indicate significant differences over time.

#### Male participants

There was a significant main effect of time on boys’ global eating disorder symptom scores (*F* [2, 1168.36] = 11.98, *p* < .001) and on rates of binge eating (Wald Χ^2^ [2] = 20.22, *p* < .001) and attempts to follow strict dietary rules (Wald Χ^2^ [2] = 17.92, *p* < .001). For global eating disorder symptom scores, there was a small but statistically significant decrease in scores from age 14 to age 17, before stable symptoms from age 17 to 20 (see Table 1). The prevalence of objective binge eating also decreased significantly from age 14 to age 17 before stable symptoms from age 17 to age 20, whilst attempts to follow strict dietary rules decreased significantly from age 14 to age 17 but then increased to approximate 14-year levels at age 20. Prevalence rates are shown in Table 1 and odds ratios from generalised estimating equations are shown in Table 3, below. By age 20, binge eating was approximately one-third as likely to occur (OR = .29) as at age 14.

**Table 3:** Odds ratios (and 95% CI) for changes in eating disorder symptoms across adolescence, in relation to time (age), 14-year depression status, and the interaction between time and 14-year depression status, for male participants

	**Binge eating**	**Purging**	**Fasting**	**Dietary rules**	**Exercise**
Time (reference = age 14):					
Age 17	0.52**	2.15	0.82	0.65**	0.90
	(0.40 – 0.68)	(0.92 – 5.06)	(0.58 – 1.15)	(0.51 – 0.84)	(0.77 – 1.06)
Age 20	0.29**	2.29 ^1^	0.87	0.91	1.08
	(0.22 – 0.37)	(1.01 – 5.16)	(0.63 – 1.19)	(0.71 – 1.17)	(0.77 – 1.06)
Depression at age 14 (reference = no depression)					
Mild or greater	2.89**	2.72**	2.80**	1.71*	2.29**
	(1.31 – 6.40)	(1.00 – 7.58)	(1.32 – 5.97)	(1.02 – 2.87)	(1.21 – 4.31)
Time x depression (reference = age 14, no depression)					
Age 17 x 14-year	0.75	0.67	1.03	0.68	1.19
depression	(0.32 – 1.79)	(0.05 – 8.58)	(0.36 – 2.89)	(0.18 – 2.50)	(0.38 – 3.68)
Age 20 x 14-year	0.67	1.53	0.41	0.92	0.74
depression	(0.24 – 1.84)	(0.11 – 10.39)	(0.10 – 1.62)	(0.24 – 3.52)	(0.26 – 2.10)

* *p* < .05 ** *p* < .01 ^1^ The overall effect of time on purging prevalence from 14 to 20 years was not statistically significant, but the comparison between 14 and 20 years was significant at *p* = .046.

*Note.* Models are adjusted for body mass index and family income. The effective sample size with these covariates was n = 678 for models with time only and n = 644 for models with depression.

There were no significant main effects of time on the prevalence of purging (Wald Χ^2^ [2] = 4.18, *p* = .123), fasting (Wald Χ^2^ [2] = 1.38, *p* = .502) or hard exercise (Wald Χ^2^ [2] = 6.06, *p* = .500) in boys.

#### Female participants

For girls, significant main effects of time were identified for all eating disorder symptoms except attempts to follow strict dietary rules (Wald Χ^2^ [2] = 5.53, *p* = .063). There was a small but statistically significant increase in global symptom scores from age 14 to age 17, before symptoms decreased to approximate 14-year levels at age 20 (*F *[2, 865.18] = 14.71, *p* < .001) (see Table 2). The same pattern was observed for fasting (Wald Χ^2^ [2] = 37.01, *p* < .001). The prevalence of binge eating was stable from age 14 to age 17 before increasing significantly to age 20 (Wald Χ^2^ [2] = 11.99, *p* < .001), whereas prevalence rates increased significantly from age 14 to ages 17 and 20 for purging (Wald Χ^2^ [2] = 48.64, *p* < .001) and hard exercise (Wald Χ^2^ [2] = 33.59, *p* < .001). Prevalence rates are shown in Table 2 and odds ratios from generalised estimating equations are shown in Table 4, below. As shown, the greatest increase in prevalence was observed for purging, with this behaviour being over 4 times as likely to occur at age 20 than at age 14 (OR = 4.23).

**Table 4:** Odds ratios (and 95% CI) for changes in eating disorder symptoms across adolescence, in relation to time (age), 14-year depression status, and the interaction between time and 14-year depression status, for female participants (n = 703)

	**Binge eating**	**Purging**	**Fasting**	**Dietary rules**	**Exercise**
Time (reference = age 14)					
Age 17	0.93	4.07**	1.84**	1.23	1.36**
	(0.78 - 1.11)	(2.69 - 6.16)	(1.49 - 2.27)	(1.00 - 1.50)	(1.15 - 1.62)
Age 20	1.22**	4.23**	1.24	0.98	1.75**
	(1.01 - 1.47)	(2.78 - 6.43)	(0.97 - 1.58)	(0.81 - 1.19)	(1.44 - 2.11)
Depression at age 14 (reference = no depression)					
Mild or greater	3.54**	3.29**	2.89**	1.25	1.40
	(2.18 - 5.75)	(2.14 - 5.09)	(1.99 - 4.18)	(0.90 - 1.73)	(0.98 - 2.01)
Time x depression (reference = age 14, no depression)					
Age 17 x 14-year	0.51**	0.39**	0.40**	0.84	0.71
depression	(0.30 - 0.88)	(0.16 - 0.97)	(0.23 - 0.70)	(0.47 - 1.50)	(0.37 - 1.36)
Age 20 x 14-year	0.41**	0.21**	0.43**	0.43*	0.48
depression	(0.21 - 0.82)	(0.08 - 1.85)	(0.22 - 0.84)	(0.28 - 0.82)	(0.24 - 0.97)

* *p* < .05 ** *p* < .01

**Figure 1:**
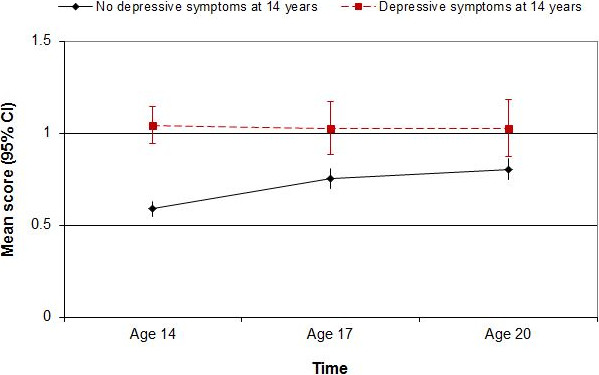
**Changes in girls’ global eating disorder symptom scores (mean scores with 95% confidence intervals), by time and 14-year depression status.** There are significant main effects of time and 14-year depression status, and a significant interaction effect between time and depression.

**Figure 2:**
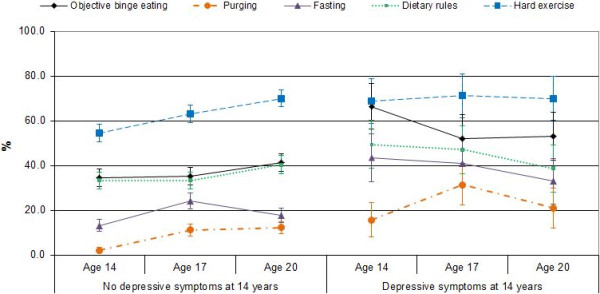
**Prevalence rates (with 95% confidence intervals) for categorical eating disorder symptoms in girls, by time and 14-year depression status.** There are significant main effects of time for binge eating, purging, fasting and driven exercise; significant main effects of 14-year depression group for binge eating, purging and fasting; and significant time x depression interaction effects for binge eating, purging, fasting and attempts to follow strict dietary rules.

*Note.* Models are adjusted for body mass index and family income. The effective sample size with these covariates was n = 697 for models with time only and n = 619 for models with depression. Interaction effects are summarised in Figures 1 and 2.

When considering differences between male and female participants, global eating disorder symptom scores and rates of categorical eating disorder symptoms were significantly higher in females than males, for all symptoms at all time points (*p* < .002). Body mass index was greater in females than in males at age 14 only (*p* = .008).

### Prospective effects of depression on eating disorder symptoms

#### Male participants

There was a significant main effect of 14-year depression status on boys’ global eating disorder symptom scores (*F* [1, 1575.17] = 32.58, *p* < .001) and on rates of binge eating (Wald Χ^2^ [1] = 6.37, *p* = .012), purging (Wald Χ^2^ [1] = 94.72, *p* < .001), fasting (Wald Χ^2^ [1] = 5.89, *p* = .015), attempts to follow strict dietary rules (Wald Χ^2^ [1] = 4.14, *p* = .042), and hard exercise (Wald Χ^2^ [1] = 6.57, *p* = .010). In all cases, symptoms were higher for boys with elevated depression at age 14, on average and across the 6-year study period, than for boys without depression at age 14. Odds ratios are shown in Table 3. Boys with elevated depressive symptoms at age 14 were nearly three times as likely to report binge eating (OR = 2.89), purging (OR = 2.72) and fasting (OR = 2.80) than boys without elevated depression.

There were no significant time x depression interaction effects for global eating disorder symptom scores (*F* [2, 918.94] = 1.71, *p* = .182), binge eating (Wald Χ^2^ [2] = 0.64, *p* = .724), purging (Wald Χ^2^ [1] = 0.96, *p* = .756), fasting (Wald Χ^2^ [2] = 2.04, *p* < .361), strict dietary rules (Wald Χ^2^ [2] = 0.35, *p* = .839), or hard exercise (Wald Χ^2^ [2] = 0.94, *p* = .623).

#### Girls

There was a significant main effect of 14-year depression status on girls’ global eating disorder symptom scores (*F* [1, 1519.48] = 68.13, *p* < .001) and on rates of binge eating (Wald Χ^2^ [1] = 19.06, *p* < .001), purging (Wald Χ^2^ [1] = 35.68, *p* < .001), and fasting (Wald Χ^2^ [1] = 32.18, *p* < .001). In all cases, symptoms were higher for girls with elevated depression at age 14, on average and across the 6-year study period, than for girls without depression at age 14. There was no significant main effect of depression on girls’ attempts to follow strict dietary rules (Wald Χ^2^ [1] = 1.77, *p* = .183) or hard exercise (Wald Χ^2^ [1] = 3.16, *p* = .076). Odds ratios are provided in Table 4, and show that girls with elevated depressive symptoms at age 14 were approximately three times as likely to report binge eating (OR = 3.54), purging (OR = 3.29) and fasting (OR = 2.89) than girls without elevated depression.

Significant time x depression interaction effects were also identified for global eating disorder symptom scores (*F* [2, 906.72] = 9.86, *p* < .001), binge eating (Wald Χ^2^ [2] = 7.34, *p* = .025), purging (Wald Χ^2^ [2] = 10.61, *p* = .005), fasting (Wald Χ^2^ [2] = 9.59, *p* = .008), and attempts to follow strict dietary rules (Wald Χ^2^ [2] = 7.04, *p* = .030). There was no significant time x depression interaction for hard exercise (Wald Χ^2^ [2] = 3.60, *p* = .165).

The interaction effect for global eating disorder symptoms is summarised in Figure 1. Girls with elevated depressive symptoms at age 14 had higher scores at each time point than girls without depressive symptoms at age 14, but scores remained stable between age 14 and age 20 for the depressive symptom group, whereas they increased over time for the non-depressed group.

Interaction effects for the categorical variables are summarised in Figure 2. Girls with depressive symptoms at age 14 had higher prevalence rates for each eating disorder symptom at each time point, with the exception of dietary rules at age 20. However, rates for binge eating, fasting, and dietary rules decreased by approximately 10% from age 14 to age 20 for this group, and rates for purging increased from at age 14 to age 17 but then decreased to approximate 14-year levels at age 20. For girls *without* depressive symptoms at age 14, rates for binge eating, purging, fasting and dietary rules increased over time (see Figure 2).

## Discussion

This study has described the prevalence and trajectory of eating disorder symptoms in male and female adolescents followed from 14 to 20 years of age. Symptom trajectories varied according to the specific eating disorder symptom studied, participant sex, and the presence of depressive symptoms in early adolescence.

For boys, eating disorder symptoms tended to be stable (for purging, fasting and hard exercise) or decreasing (for global eating disorder symptom scores and binge eating) from 14 to 17 years, and then stable to 20 years. An exception was found for strict dietary rules, where prevalence decreased from age 14 to age 17 before increasing to baseline levels by age 20. These results are consistent with findings from the small number of previous studies with adolescent males [3,5,8], which observed stable or reducing symptoms from early to middle adolescence, before an increase in symptoms in late adolescence or early adulthood. In this sample, we did not observe marked increases in eating pathology in late adolescence, suggesting that any increases in this cohort did not occur until after age 20. It is interesting to note that the one symptom that *did* increase in late adolescence was attempts to follow strict dietary rules. Cognitive-behavioural theories would suggest that an increase in strict dieting may precede a subsequent increase in binge eating, and possibly purging [22,49]. This allows for the hypothesis that male participants in this sample are at risk for these behaviours as they move in to early adulthood.

Boys who reported depressive symptoms at age 14 had higher rates of all eating disorder symptoms, on average, than boys who did not report marked depression at age 14. However, depression at age 14 did not relate significantly to symptom trajectories over the following 6 years. If males do experience increases in eating disorder symptoms later than females, as they transition into adulthood, it is possible that depressive symptoms would be more relevant to male symptom trajectories if assessed at a later time point. Future studies may need to time male assessments accordingly, to capture changes in pathology after age 20. As relatively few studies have tested prospective associations between depression and eating pathology in males [21,23], it also seems possible that these relationships are less pronounced for males than for females. If so, it would be important to identify other factors that may influence the onset and persistence of male eating pathology.

For girls, the prevalence of strict dietary rules remained stable from 14 to 20 years but rates for all other symptoms varied significantly over time. Fasting and global eating disorder symptom scores increased from age 14 to peak in prevalence at age 17, before returning to 14-year levels at age 20. Rates of binge eating were stable from age 14 to age 17 and increased significantly thereafter, rising by approximately 6% before age 20. In contrast, rates of purging and hard exercise increased by approximately 10% from age 14 to age 17, and then remained stable through to age 20. These findings are broadly consistent with those reported elsewhere [e.g., [3,5,6], but the sequence of binge eating relative to purging was unexpected. Others have observed peaks in binge eating risk prior to peaks in purging [6,20], an order that is consistent with theories of BN development and the view that purging is a compensatory response to binge eating behaviour [49]. However, Neumark-Sztainer et al. [5] also found stable rates of binge eating in their sample of female adolescents followed from early to late adolescence, whereas rates of purging behaviour increased over that time frame. As some studies have focused on risk for symptom onset at an individual level (e.g., [6,20]), rather than changes in prevalence at a population level ([5], this study), it is possible that methodological differences may partially account for this cross-study variation. Nonetheless, additional research is indicated to clarify the relative prevalence of binge eating and purging at different time points in adolescence.

Girls’ eating disorder symptom trajectories also varied significantly according to the presence or absence of depressive symptoms at age 14. On average, girls with elevated depression reported higher rates of binge eating, purging and fasting than girls without elevated depression, consistent with previous reports [19]. However, girls with elevated depression at age 14 showed decreases in binge eating, fasting and dietary rules from 14 to 20 years, whilst girls without elevated depression at age 14 showed increases in these symptoms over time. Contrasting results were observed for purging, which was the only eating disorder symptom to increase in prevalence after age 14 for the group who reported depressive symptoms at baseline. Previous studies have found depressive symptoms to predict increases in binge eating as well as purging [20], so our failure to replicate this relationship should be followed up in future research. Attention to methodology is also important here, as we considered the effects of depression at one time point on eating disorder symptom trajectories thereafter. This is different to considering reciprocal relationships between depression and eating pathology over time, which has been the focus in several past investigations (e.g., [17]). This study tested one aspect of the putative relationship between depression and eating pathology, by considering how early adolescent depression influenced later changes in eating disorder symptoms. We do not report on reverse or reciprocal relationships (i.e., the effects of eating disorder symptoms on depression) or on associations between late adolescent depression and eating disorder symptoms.

These results have four key implications for eating disorder prevention and intervention efforts in adolescence. First, boys’ eating disorder symptoms appear to decrease in frequency in middle adolescence, suggesting that prevention and early intervention initiatives for males may be more beneficial in the period of transition to young adulthood, rather than earlier in adolescence. Second, the marked increase in purging behaviour in girls from age 14 to age 17 suggests that purging should be a focus of eating disorder screening and intervention programmes for early and middle adolescent females. Third, and consistent with previous findings [5,12], binge eating, strict dieting and driven exercise are reported by at least one in three female adolescents, regardless of age. There is a pressing need to identify effective and universal prevention, screening and intervention strategies for these behaviours. Fourth, the presence of depression in early adolescence does not appear to predict later increases in eating pathology, but it may be a marker of existing eating disorder symptoms. The reverse may also hold true, and joint assessment of depression and eating disorder symptoms in adolescents may be worthwhile. Similarly, coordinated intervention efforts for depression and eating pathology may be of benefit to some adolescents.

This study benefited from prospective, population-based data from male and female participants, attention to the full range of eating disorder symptoms, and attention to the effects of early adolescent depression on eating disorder symptom trajectories. Two limitations deserve note, however, including participant attrition within the Raine Study cohort and the lack of follow-up into adulthood. Regarding attrition, the loss of disadvantaged families is a well-replicated phenomenon in longitudinal cohort studies [47]. In the Raine Study, attrition has served to increase the representativeness of the cohort over time, as the study initially over-sampled socially-disadvantaged women. Previous analyses have shown that participants who remained in the study to adolescence are broadly comparable to the Western Australian population on a range of socio-demographic indicators [50]. Despite this, replication of our results in other cohorts is important and would help to strengthen the findings observed here. We also found that, even amongst participants who remained in the study to adolescence, individuals who completed one adolescent assessment (and who were consequently excluded from this study) were more socially disadvantaged in childhood than those who completed two or more assessments. Our results should be interpreted with this in mind, and with the understanding that different findings may emerge with a socially disadvantaged sample. Regarding follow-up duration, this issue is particularly relevant to male participants, who may be expected to show increases in eating pathology after age 20. The benefits of following male participants into early adulthood should be considered and planned for in future analyses.

## Conclusions

This study provides new data on the prevalence and trajectory of eating disorder symptoms in males and females followed from 14 to 20 years of age. Results highlight the importance of attending to sex and depressive symptoms when considering eating disorder symptom trajectories, and confirm that different eating disorder symptoms may be expected to show different trajectories across adolescence. In this sample, females showed peaks in fasting and the global index of dietary restraint and eating, weight and shape concerns at age 17. Binge eating peaked at age 20, whilst purging and hard exercise for weight control increased between ages 14 and 17 and remained elevated at age 20. For males, global eating disorder symptom scores and binge eating decreased across adolescence, whilst the prevalence of strict dietary rules reduced from age 14 to age 17 before returning to baseline levels at age 20. Depressive symptoms at age 14 impacted on eating disorder symptom trajectories in females, but not in males.

## Consent

Written informed consent was obtained from participants for their data to be used for research purposes, including publications.

## Appendix A

Eating disorder questionnaire items (adapted from the Child Eating Disorder Examination [30] and Eating Disorder Examination-Questionnaire [31]).

### General instructions

• **Please read each question carefully.** Circle the most appropriate option.

• **Please take your time.** If you are uncomfortable about a question or unsure of an answer, please leave it blank and contact one of the Raine Study staff.

• **Remember all answers are STRICTLY confidential.**

### Over the last four weeks (one month)…

Q1. Have you been trying hard to eat less to change your shape or weight? (even if you haven’t managed to do so)

Not at all 0

Some of the time (once per week / a few times a month) 1

A lot of the time (a few times a week) 2

Most of the time (every day or nearly every day) 3

Q2. Have you gone for long periods of time (8 hours or more) without eating anything to try and change your shape or weight?

Not at all 0

Some of the time (once per week / a few times a month) 1

A lot of the time (a few times a week) 2

Most of the time (every day or nearly every day)3

Q3. Have you tried not to eat certain foods (like chocolate or chips) to try to change your shape or weight? (even if you haven’t been able to do so)

Not at all 0

Some of the time (once per week / a few times a month) 1

A lot of the time (a few times a week) 2

Most of the time (every day or nearly every day)3

Q4. Have you tried to stick to any definite rules about diet or eating? For example sticking to a calorie limit, a set amount of food or rules about what or when you should eat? (even if you haven’t been able to do so)

Not at all 0

Some of the time (once per week / a few times a month) 1

A lot of the time (a few times a week) 2

Most of the time (every day or nearly every day) 3

Q5. Have you been thinking about food or calories so much that you’ve found it hard to concentrate on things you are interested in? For example reading, watching TV or following a conversation?

Not at all0

Some of the time (once per week / a few times a month) 1

A lot of the time (a few times a week) 2

Most of the time (every day or nearly every day) 3

Q6. Have you been afraid of losing control over your eating?

Not at all 0

Some of the time (once per week / a few times a month)1

A lot of the time (a few times a week) 2

Most of the time (every day or nearly every day)3

Q7. Have there been times when you felt you had eaten an unusually large amount of food – much more than what most people would eat in the same situation?

Not at all 0

Some of the time (once per week / a few times a month) 1

A lot of the time (a few times a week) 2

Most of the time (every day or nearly every day) 3

Q8. Have you felt that you couldn’t control what or how much you were eating?

Not at all 0

Some of the time (once per week / a few times a month) 1

A lot of the time (a few times a week) 2

Most of the time (every day or nearly every day) 3

Q9. Have you felt that you couldn’t stop eating once you had started?

Not at all 0

Some of the time (once per week / a few times a month) 1

A lot of the time (a few times a week) 2

Most of the time (every day or nearly every day)3

Q10. Have you felt guilty after eating?

Not at all 0

Some of the time (once per week / a few times a month) 1

A lot of the time (a few times a week) 2

Most of the time (every day or nearly every day) 3

Q11. Have you eaten in secret because you are embarrassed by how much you eat?

Not at all 0

Some of the time (once per week / a few times a month) 1

A lot of the time (a few times a week) 2

Most of the time (every day or nearly every day) 3

Q12. Have you been afraid that you might gain weight or become fat?

Not at all 0

Some of the time (once per week / a few times a month) 1

A lot of the time (a few times a week) 2

Most of the time (every day or nearly every day) 3

Q13. Have you felt fat?

Not at all 0

Some of the time (once per week / a few times a month) 1

A lot of the time (a few times a week) 2

Most of the time (every day or nearly every day) 3

Q14. Have you had a strong desire to lose weight?

Not at all 0

Some of the time (once per week / a few times a month) 1

A lot of the time (a few times a week) 2

Most of the time (every day or nearly every day) 3

Q15. Have you made yourself sick (vomit) after eating to control your weight?

Not at all 0

Some of the time (once per week / a few times a month) 1

A lot of the time (a few times a week) 2

Most of the time (every day or nearly every day) 3

Q16. Have you taken any pills (like laxatives or water pills) to try to control your weight?

Not at all 0

Some of the time (once per week / a few times a month) 1

A lot of the time (a few times a week) 2

Most of the time (every day or nearly every day) 3

Q17. Have you exercised hard to control your weight?

Not at all 0

Some of the time (once per week / a few times a month) 1

A lot of the time (a few times a week) 2

Most of the time (every day or nearly every day) 3

People have different ideas about what sort of things are important to them in how they think about themselves. For some people doing well at school is very important to them in how they think about themselves, for others, how they are getting on with friends is very important.

We’re now going to ask you to think about how important weight and shape are to you in how you think of yourself as a person.

Q18. Has your weight been important in how you think of yourself as a person?

Not at all 0

Some of the time (once per week / a few times a month) 1

A lot of the time (a few times a week) 2

Most of the time (every day or nearly every day) 3

Q19. Has your shape been important in how you think of yourself as a person?

Not at all 0

Some of the time (once per week / a few times a month) 1

A lot of the time (a few times a week) 2

Most of the time (every day or nearly every day) 3

Q20. Have you felt unhappy about your weight?

Not at all 0

Some of the time (once per week / a few times a month) 1

A lot of the time (a few times a week) 2

Most of the time (every day or nearly every day) 3

Q21. Have you felt unhappy about your shape?

Not at all 0

Some of the time (once per week / a few times a month) 1

A lot of the time (a few times a week) 2

Most of the time (every day or nearly every day) 3

Q22. Have you been concerned about other people seeing you eat?

Not at all 0

Some of the time (once per week / a few times a month) 1

A lot of the time (a few times a week) 2

Most of the time (every day or nearly every day) 3

Q23. Have you felt uncomfortable about seeing your body, for example, in the mirror, or in the bath or shower?

Not at all 0

Some of the time (once per week / a few times a month) 1

A lot of the time (a few times a week) 2

Most of the time (every day or nearly every day) 3

Q24. Have you felt uncomfortable about other people seeing your body, for example, in the change rooms, or when wearing bathers or tight clothes

Not at all 0

Some of the time (once per week / a few times a month) 1

A lot of the time (a few times a week) 2

Most of the time (every day or nearly every day) 3

## Abbreviations

BDI-Y: Beck depression inventory-youth; BMI: Body mass index; ChEDE: Child eating disorder examination; EDE-Q: Eating disorder examination; KEMH: King edward memorial hospital for women; Raine study: Western australian pregnancy cohort (Raine) study.

## Competing interests

The authors declare that they have no competing interests.

## Authors’ contributions

KA, WO and SB participated in the design of the study. RC provided guidance on the statistical analyses. KA performed the statistical analyses and drafted the manuscript. All authors read and approved the final manuscript.
